# Performance Analysis of Relay Subset Selection for Amplify-and-Forward Cognitive Relay Networks

**DOI:** 10.1155/2014/548082

**Published:** 2014-03-06

**Authors:** Kiran Sultan, Ijaz Mansoor Qureshi, Aqdas Naveed Malik, Muhammad Zubair

**Affiliations:** ^1^Department of Electrical Engineering, Air University, Islamabad, Pakistan; ^2^Department of Electronics Engineering, IIU, Islamabad, Pakistan

## Abstract

Cooperative communication is regarded as a key technology in wireless networks, including cognitive radio networks (CRNs), which increases the diversity order of the signal to combat the unfavorable effects of the fading channels, by allowing distributed terminals to collaborate through sophisticated signal processing. Underlay CRNs have strict interference constraints towards the secondary users (SUs) active in the frequency band of the primary users (PUs), which limits their transmit power and their coverage area. Relay selection offers a potential solution to the challenges faced by underlay networks, by selecting either single best relay or a subset of potential relay set under different design requirements and assumptions. The best relay selection schemes proposed in the literature for amplify-and-forward (AF) based underlay cognitive relay networks have been very well studied in terms of outage probability (OP) and bit error rate (BER), which is deficient in multiple relay selection schemes. The novelty of this work is to study the outage behavior of multiple relay selection in the underlay CRN and derive the closed-form expressions for the OP and BER through cumulative distribution function (CDF) of the SNR received at the destination. The effectiveness of relay subset selection is shown through simulation results.

## 1. Introduction

Enabling secondary transmissions ensuring minimum quality of service (QoS) with constrained transmission power is a major design challenge faced by underlay CRNs and it requires fine tuning and adjustment of the transmit power of the SUs. The purpose of limiting the transmit power is to keep the primary communication undisturbed [1]. In [2], the authors suggested a transmit power allocation scheme for dual-hop CRNs operating in AF mode, under transmit power constraints and interference constraints. First, the optimization problem was simplified by relaxing the transmit power constraint to obtain a suboptimal solution, which was then further utilized to propose a power allocation scheme in order to satisfy both constraints all the time.

The problem highlighted above becomes more complicated when the secondary source-destination pair is unable to communicate directly due to deep fading or shadowing and so forth. Cooperative communication [3] is an effective means of increasing the spatial diversity of a signal in wireless communication networks. Such a communication strategy efficiently improves system throughput, combats channel fading, reduces power consumption, and increases transmission reliability and coverage area [4–6]. Cooperative communication techniques follow such approaches as collaborative signal processing, cooperative coding, and relaying [7]. The literature review reveals that amplify-and-forward (AF) is the simplest and the most widely employed relaying protocol, in which the relay just scales the received message and forwards it to the destination without performing any regenerating action, thus requiring less processing and low power consumption at the relay [8]. Relay-assisted CRNs have emerged as a potential solution to cope with the challenges faced by underlay networks. However, it may not be a feasible idea to use all the relays in a cognitive radio system to assist SUs, because the interference produced by the relays may exceed the interference threshold of the PUs, which forces the secondary network to transmit at very low power, reducing the signal-to-noise ratio (SNR) at the destination. Relay(s) selection comes as a fascinating solution to this problem; however, selection of subset of multiple relays satisfying interference and transmit power constraints is more complex than single relay selection in underlay networks and limited effort has been done in this context so far. The interference threshold can be defined by average or instantaneous interference power received at the primary receiver [9]. The instantaneous or peak interference power requires knowledge about instantaneous channel gains of the interference channels and it is suitable for real-time traffic. The average interference power applies to nonreal time traffic where the average SNR determines the QoS.

Some of the research contributions in the area of best relay selection are as follows. In [10], Fredj and Aïssa presented a scenario in which a secondary transmitter used the services of intermediate relays to communicate to its receiver. In this scenario, best relay was selected from the potential relay set to enable secondary communication under interference constraints. Furthermore, end-to-end SNR statistics were derived and bit error rate (BER) was evaluated for different modulation schemes. In [11], Seyfi et al. proposed a best relay selection scheme for dual-hop cognitive relay network under transmit power constraints and interference constraints. Furthermore, the outage probability of the secondary network with relay selection was derived while considering the effect of PU interference. The derived results were tested through simulations. In [12], Bao et al. proposed best relay selection and considered tight lower bound of the end-to-end SNR to derive the closed-form expressions for cumulative distribution function (CDF) and probability density function (PDF) over nonidentical Rayleigh fading channels. The derived results were used to investigate the outage probability and average symbol error probability of proposed system. The performance was evaluated against some key parameters. The asymptotic analysis of the scenario showed that interference constraint does not affect the diversity gain. Li investigated best relay selection based on full and partial channel state information (CSI) in [13] and compared the performance of both schemes by deriving the closed-form expressions for outage probability. For this purpose, a cluster of cognitive relays assisting a single source-destination pair was considered. It was proved that partial-CSI-based relay selection was outperformed by the full-CSI-based relay selection.

Research contributions in the area of multiple relay selection are, however, quite limited. In our correspondence, we will use “relay subset selection” or “multiple relay selection” interchangeably. Multiple relay selection schemes to maximize the SNR at the destination in an underlay CRN were proposed in [14], and their performance was compared against different levels of source transmit power, considering different sizes of potential relay network and different interference threshold levels. Naeem et al. considered a dual-hop CRN and proposed a multiple relay selection scheme with interference awareness for underlay CR systems in [15]. It was proved through simulations that the performance of the proposed scheme approached exhaustive search technique while having low implementation complexity.

These prior works have significantly improved our understanding of relay-assisted CRNs and are selected for discussion because all the contributions were built on some common assumptions which are as These prior contributions have significantly improved our understanding of relay-assisted CRNs, and all of these were built on some common assumptions which are explained as follows. First, underlay spectrum sharing model was assumed for each scenario. Second, all schemes assumed severe shadowing on the line-of-sight path between source-destination pair, thus making direct communication impossible. Third, all system models were built up using single-antenna terminals. Fourth, AF relaying was assumed at the cognitive relay network. Fifth, all the highlighted contributions for best and multiple relay selection assume the availability of CSI of the interference channels. Each of the proposed schemes has been analyzed with interference and transmit power constraints. Furthermore, these contributions have been highlighted due to the reason that performance analysis in terms of outage behavior and BER has been carried out only for single (best) relay selection schemes. However, the effect of multiple relay selection on the OP and BER of the secondary system operating in an underlay spectrum sharing environment is not presently available in the literature, to the best of our knowledge.

In this paper, the deficiencies highlighted in the performance evaluation of multiple relay selection schemes have been focused on. This paper investigates, for the first time, the outage behavior and BER of the secondary network for multiple relay selection, which has not been done so far, to the best of our knowledge, for AF based underlay cognitive relay networks. Furthermore, similar derivation has been carried out for the single best relay selection scheme for fair comparison. A dual-hop relay-assisted CRN is considered for this purpose, and a multiple relay selection scheme is proposed, aiming to maximize the secondary system performance, while satisfying the interference threshold of the primary network. We have carried out the comparison between the proposed multiple relay selection scheme and the best relay selection scheme and proved that the multiple relay selection outperforms the best relay selection in terms of OP and BER.

The remaining paper is structured as follows. The system model and the mathematical formulation of the problem have been explained in Section 2. The algorithm proposed for multiple relay selection is explained in Section 3. The performance analysis has been carried out in Section 4 followed by Section 5 which presents the simulation results. The whole work is concluded in Section 6.

## 2. System Model and Problem Formulation

**Figure 1:**
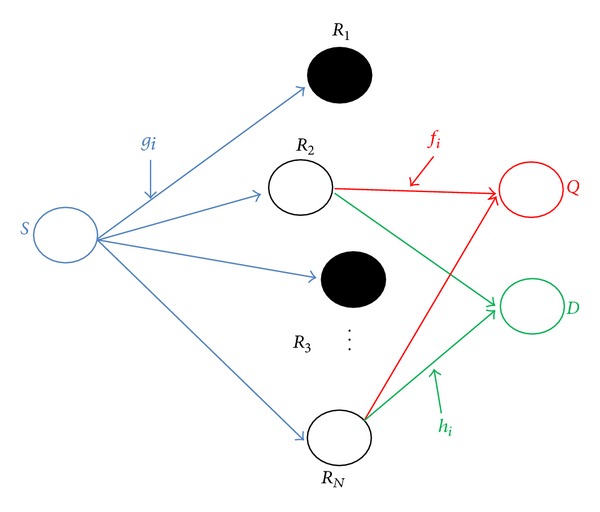
The system model.

Figure 1 shows the system model comprising a secondary source *S*, destination *D*, and a PU *Q*. The end-to-end secondary communication is entirely dependent on a potential relay set consisting of *N* candidates having cognitive radio capabilities due to a large physical separation involved between source-destination pair. The entire relay network exists near a PU in an underlay spectrum sharing mode, while the source being far away from the PU does not interfere with the primary signals.

Rayleigh flat-fading scenario is assumed, in which the independent and identically distributed (i.i.d.) source-relay, relay-destination, and relay-PU channel coefficients are designated as {*g*_*i*_}_*i*=1_^*N*^, {*h*_*i*_}_*i*=1_^*N*^, and {*f*_*i*_}_*i*=1_^*N*^, respectively, where *f*_*i*_ is treated as the interference channel. It is further assumed that the instantaneous CSI is available at each potential relay, and the instantaneous value of interference threshold is computed to perform relay selection. Single-antenna terminals are assumed at the primary and secondary networks, and relays employ AF protocol with adjustable gains. The half-duplex mode of communication takes place at the relay network which is completed in two time slots. The source transmits a symbol *s* in time slot 1 and the received signal at the *i*th relay is given as

(1) $$\begin{array}{c} y_{1i}=\sqrt{P_{s}}g_{i}s+\eta_{1i}, \end{array}$$

where *P*_*s*_ denotes the source transmit power and *η*_1*i*_ represents additive white Gaussian noise (AWGN) at the *i*th relay with zero mean and variance *N*_0_. In time slot 2, the destination receives the scaled version of the received message from the relay network while the source is silent. The signal *y*_*D*_ received at the destination *D* is expressed as

(2) $$\begin{array}{c} y_{D}=y_{1i}^{\prime }h_{i}+\eta_{D}, \end{array}$$

where *η*_*D*_ is modeled as AWGN with variance *N*_0_, received at the destination. The signal *y*_1*i*_′ amplified according to AF scheme is given as

(3) $$\begin{array}{c} y_{1i}^{\prime }=\sqrt{\frac{P_{i}}{P_{s}{\left|g_{i}\right|}^{2}+N_{0}}}y_{1i}, \end{array}$$

where *P*_*i*_ represents the transmit power of the *i*th relay in the above equation and is defined in AF relaying as

(4) $$\begin{array}{c} P_{i}=A_{i}^{2}\left(P_{s}{\left|g_{i}\right|}^{2}+N_{0}\right), \end{array}$$

where *A*_*i*_ is the randomly selected amplification factor of the *i*th relay according to the proposed algorithm.

Substituting (1) and (3) in (2) and solving the resulting expression, end-to-end SNR *γ*_*R*_*i*__ of the *i*th relay link can be expressed as [16]

(5) $$\begin{array}{c} \gamma_{R_{i}}=\frac{P_{s}{\left|g_{i}\right|}^{2}P_{i}{\left|h_{i}\right|}^{2}}{1+P_{s}{\left|g_{i}\right|}^{2}+P_{i}{\left|h_{i}\right|}^{2}}. \end{array}$$

Or in compact form

(6) $$\begin{array}{c} \gamma_{R_{i}}=\frac{\gamma_{1i}\gamma_{2i}}{1+\gamma_{1i}+\gamma_{2i}}, \end{array}$$

where *γ*_1*i*_ = *P*_*s*_|*g*_*i*_|^2^ and *γ*_2*i*_ = *P*_*i*_|*h*_*i*_|^2^ denote the SNR achieved at the source-relay and relay-destination links, respectively, with noise variance normalized to one.

The end-to-end SNR at the secondary destination due to *N* relaying links is then given by

(7) $$\begin{array}{c} \gamma_{D}=\overset{N}{\underset{i=1}{\sum }}\gamma_{R_{i}}=\overset{N}{\underset{i=1}{\sum }}\frac{\gamma_{1i}\gamma_{2i}}{1+\gamma_{1i}+\gamma_{2i}}. \end{array}$$

We explain our proposed relay subset selection problem as follows. Let $\overset{-}{P}$ represent the transmit power vector of the potential relays in the network; that is, $\overset{-}{P}=[P_{1},P_{2},P_{3},\ldots ,P_{N}]$. The number of all nontrivial subsets $\Omega \subset \overset{-}{P}$ is given by $M=\sum_{k=1}^{N}\left(\begin{array}{c} N\\ k \end{array}\right)$. The *l*th subset is denoted as *Ω*_*l*_, where *l* ∈ {1,2,…, *M*}. The cardinality of *l*th subset is *N*_*l*_. Next is to compute total interference power due to each *l*th subset of relays towards the PU, where interference offered by each *i*th relay in any subset is defined as *I*_*i*_ = *P*_*i*_|*f*_*i*_|^2^.

Let *J* be the number of subsets out of *M*, denoted as {*L*_*j*_}_*j*=1_^*J*^, which satisfy the interference threshold *ζ* towards the PU. The interference constraint for *j*th such subset can be given as

(8) $$\begin{array}{c} I^{j}=\overset{}{\underset{i\in L_{j}}{\sum }}I_{i}^{}=\underset{i\in L_{j}}{\sum }P_{i}^{}{\left|f_{i}\right|}^{2}\leq \zeta \;j=1,2,\ldots ,J. \end{array}$$

The mathematical formulation of this optimization problem is as follows:

(9) $$\begin{array}{c} \underset{L_{j}}{\max}\left[\gamma_{D}=\overset{}{\underset{i\in L_{j}}{\sum }}\gamma_{R_{i}}=\overset{}{\underset{i\in L_{j}}{\sum }}\frac{\gamma_{1i}\gamma_{2i}}{1+\gamma_{1i}+\gamma_{2i}}\right], \end{array}$$

satisfying the constraint *I* ^*j*^ ≤ *ζ*

In order to investigate the performance of the overall system in terms of outage probability and average probability of error, we need to know the distribution of *γ*_*D*_ which is not mathematically tractable. To overcome this problem, tight upper and lower bounds for *γ*_*R*_*i*__ in (6) exist in the literature [17]; that is, *γ*_*R*_*i*__^*lb*^ ≤ *γ*_*R*_*i*__ < *γ*_*R*_*i*__^*ub*^, where  *γ*_*R*_*i*__^*ub*^ = min⁡(*γ*_1*i*_, *γ*_2*i*_) and *γ*_*R*_*i*__^*lb*^ = (1/2)min⁡(*γ*_1*i*_, *γ*_2*i*_). The bounds on *γ*_*R*_*i*__ show that the minimum value of *γ*_*R*_*i*__ occurs when *γ*_2*i*_ = *γ*_1*i*_, and, for this case, *γ*_*R*_*i*__ = *γ*_1*i*_/2, and, if *γ*_2*i*_ is increased further through transmit power control, the upper bound is approached. Keeping the behavior of *γ*_*R*_*i*__ under consideration, we aim to maximize *γ*_2*i*_ through controlled transmit power allocation to each relay so that *γ*_*R*_*i*__ of each relay link tends to approach its upper bound causing an overall favorable impact on *γ*_*D*_ = ∑_*i*∈*L*_*j*__ *γ*_*R*_*i*__, while keeping the sum interference constraint satisfied. Thus, the relay subset selection algorithm aims to pick up that subset of relays, which maximizes combined SNR of relay-links, *γ*_*j*_, where, for each *j*th subset *L*_*j*_, *γ*_*j*_ is defined as *γ*_*j*_ = ∑_*i*∈*L*_*j*__ *γ*_2*i*_.

Thus, the mathematically tractable form of our optimization problem is given as

(10) max⁡Lj [γj=∑i∈Ljγ2i]s.t. Ij≤ζ.

## 3. The Proposed Algorithm

**Figure 2:**
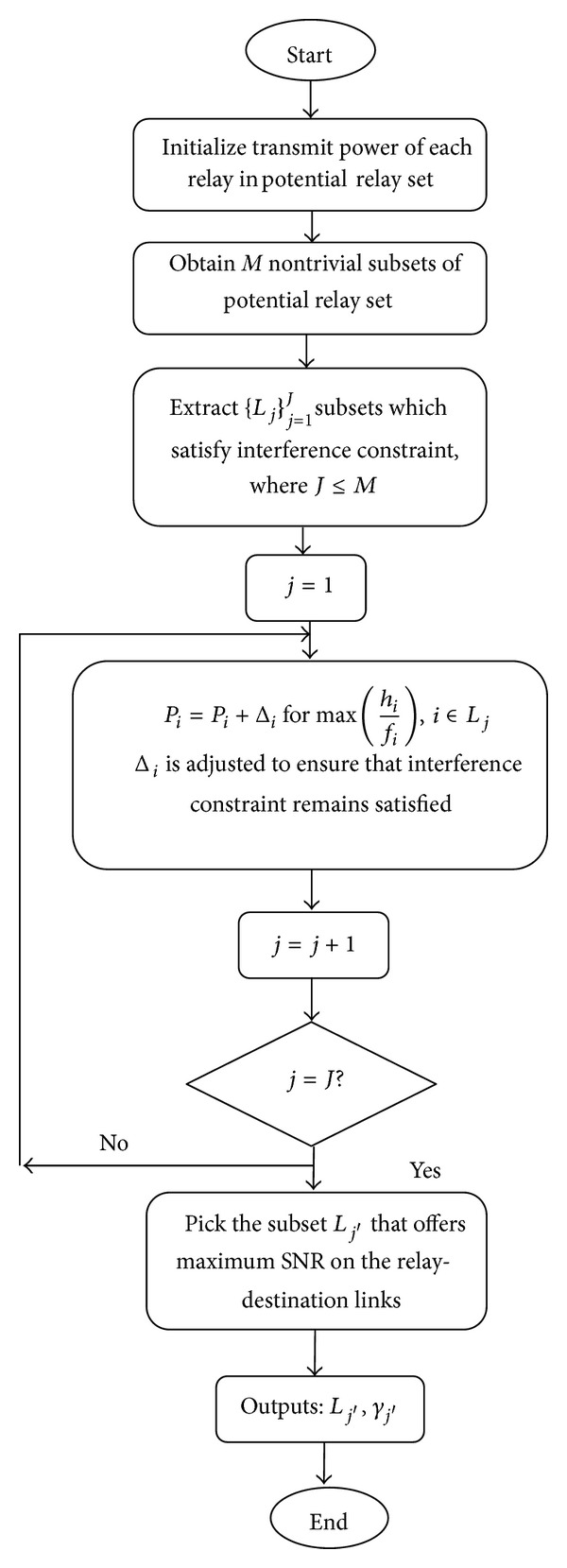
Flowchart.

Let Γ = {1,2,…, *N*} be the initial set of potential relays. The proposed algorithm works as follows. Transmit power of each relay is initialized, followed by selecting all possible subsets of relays which are able to satisfy the sum interference power threshold *ζ* set by the PU. For all such subsets, combined SNR of relay-destination links is computed and finally that subset is declared as the selected subset which maximizes the SNR. The pseudocode is provided in Algorithm 1. For more clarity, the flowchart of the proposed algorithm is also presented in Figure 2.

## 4. Performance Analysis

In this section, the performance of the proposed multiple relay selection scheme is investigated and has been compared with the best relay selection scheme. The criterion for relay selection is kept the same for both schemes for fair comparison. Performance evaluation is carried out in terms of outage probability and average probability of error. We consider both cases separately as follows.

### 4.1. Multiple Relay Selection

As mentioned earlier, Rayleigh distributed channel coefficients are assumed for the considered network with their squared amplitudes being exponential random variables. Therefore, the PDFs of *γ*_2*i*_ and *I*_*i*_, being independent and exponentially distributed, are given by

(11a) $$\begin{array}{c} p_{\gamma_{2i}}\left(\gamma \right)=\frac{1}{\alpha_{i}}e^{-\gamma /\alpha_{i}}{,\;\;p}_{I_{i}}\left(x\right)=\frac{1}{\beta_{i}}e^{-x/\beta_{i}}\;i\in {\left[L_{j}\right]}_{j=1}^{J} \end{array}$$

and the corresponding CDFs are given by

(11b) $$\begin{array}{c} P_{\gamma_{2i}}\left(\gamma \right)=1-e^{-\gamma /\alpha_{i}},\;\;P_{I_{i}}\left(x\right)=1-e^{-x/\beta_{i}},\;i\in {\left[L_{j}\right]}_{j=1}^{J}, \end{array}$$

where *α*_*i*_ denotes the average second-hop SNR for *i*th relaying link and *β*_*i*_ is the average strength of interference channel from the *i*th relay and PU.

Given *J* subsets for selection, the conditional PDF of the SNR of the finally chosen subset *γ*_sel_ according to the proposed relay subset selection scheme where *γ*_sel_ ∈ *L*_*j*_ is given as

(12) pγsel(γ ∣ J)=pγ1(γ)Pr[γ1>γ2]⋯Pr[γ1>γJ]+pγ2(γ)Pr[γ2>γ1]⋯Pr[γ2>γJ]+⋯+pγJ(γ)Pr[γJ>γ1]⋯Pr[γJ>γJ−1].

In order to simplify further analysis of above PDF, we assume that, during a hop transmission, instantaneous SNRs have the same average values for all relays. Hence, *α*_1_ = *α*_2_ = ⋯ = *α*_*N*′_ = *α*. Using this assumption, (12) is rewritten as

(13) pγsel(γ ∣ J)=Jpγj′(γ)Pr[γj′>γj]J−1=Jpγj′(γ)Pr[γj<γj′]J−1 j≠j′.

In the above equation, the first part *p*_*γ*_*j*′__(*γ*) is the PDF of combined SNR of final selected subset being evaluated at *γ*. Since each element in the selected subset *L*_*j*′_ is exponentially distributed, then the PDF of the combined SNR *γ*_*j*′_, being the sum of exponential random variables with same mean, will be Erlang distributed and is given by

(14) $$\begin{array}{c} p_{\gamma_{j^{\prime }}}\left(\gamma \right)=\frac{1}{\alpha }\frac{e^{-\gamma /\alpha }}{\Gamma \left(N_{j^{\prime }}\right)}{\left(\frac{\gamma }{\alpha }\right)}^{N_{j^{\prime }}-1}, \end{array}$$

where *N*_*j*′_ is the cardinality of selected set *L*_*j*′_.

The second part of (13), that is, Pr[*γ*_*j*_ < *γ*_*j*′_], is the CDF of SNR of *j*th subset *γ*_*j*_ being evaluated at *γ*_*j*′_. As mentioned above, the SNR of each *j*th subset follows Erlang distribution; thus the CDF of *γ*_*j*_ will be expressed as

(15) Pγj(γj′)=Pr[γj<  γj′]=1−e−γj′/α∑n=0Nj−11n!(γj′α)n.

Therefore, the PDF of selected relay subset in (13) will take the form of

(16) pγsel(γ ∣ J)=Je−γ/αγNj′−1αNj′Γ(Nj′)×{1−e−γj′/α∑n=0Nj−11n!(γj′α)n}J−1.

An important consideration is that the PDF given above is conditioned over *J*, that is, the number of subsets which are able to satisfy the interference constraints. The value of *J* may vary from 0 to *M*. If *J* = 0, communication between secondary source-destination pair is not possible. This situation occurs if sum interference threshold *ζ* imposed by PU is too low that no subset of relays is able to meet the requirement without amplification, thus making secondary communication impossible. But this is not the case in our scenario as the relay network is assumed to be far away from the PU. Thus, *J* takes the values between 1 and *M*. If *J* = 1, there would be no relay subset selection and if *J* ≥ 2, the destination will decide which relay subset is the one satisfying the proposed criteria. The interference constraint *ζ* can be satisfied by each subset in *Ω* with a probability *P*_*ζ*_, where *P*_*ζ*_ = Pr(*I* ^*j*^ ≤ *ζ*) dictates the Erlang distribution following the same assumption for interference strengths of relayed links; that is, *β*_1_ = *β*_2_ = ⋯ = *β*_*J*_ = *β*. Thus, the PDF can be obtained in the same way as

(17) $$\begin{array}{c} p_{I^{j}}\left(I\right)=\frac{1}{\beta }\frac{e^{-I/\beta }}{\Gamma \left(N_{j}\right)}{\left(I\right)}^{N_{j}-1}. \end{array}$$

And the corresponding CDF is written as

(18) $$\begin{array}{c} P_{\zeta }=\text{Pr}\left[I^{j}<\zeta \right]=1-e^{-\zeta /\beta }\overset{N_{j}-1}{\underset{n=0}{\sum }}\frac{1}{n!}{\left(\frac{\zeta }{\beta }\right)}^{n}. \end{array}$$

Thus, the probability of availability of *J* subsets out of *M* subsets which satisfy interference threshold follows binomial distribution

(19) $$\begin{array}{c} p_{J}\left(J;M,P_{\zeta }\right)=\left(\begin{array}{c} M\\ J \end{array}\right){\left(P_{\zeta }\right)}^{J}{\left(1-P_{\zeta }\right)}^{M-J}. \end{array}$$

The unconditional PDF of SNR at the destination *γ*_sel_ due to selected subset can be found by using (18) and (19) in (16) as

(20) pγsel(γ;Ij≤ζ)=∑J=1M(MJ)J(Pζ)J(1−Pζ)M−J×{1−e−γj′/α∑n=0Nj−11n!(γj′α)n}J−1×e−γ/ααNj′Γ(Nj′)γNj′−1.

The corresponding CDF can be obtained by integrating the above PDF w.r.t. *γ* and using [18, Equation (3.381.1)]; thus,

(21) Pγsel(γ)=1Γ(Nj′)×∑J=1M(MJ)J(Pζ)J(1−Pζ)M−J×{1−e−γj′/α∑n=0Nj−11n!(γj′α)n}J−1×γ~(Nj′,γα),

where $\tilde{\gamma }(a,x)$ denotes the incomplete gamma function given in [18, Equation (8.354.1)] as

(22) $$\begin{array}{c} \tilde{\gamma }\left(a,x\right)={\overset{\infty }{\underset{k=0}{\sum }}\frac{{\left(-1\right)}^{k}}{k!\left(N_{j^{\prime }}+k\right)}\left(\frac{\gamma }{\alpha }\right)}^{N_{j^{\prime }}+k}. \end{array}$$

The outage event occurs in a communication system if the SNR received at the destination falls below a set threshold *γ*_th_. The probability of this event can be directly obtained from the CDF of the received SNR given in (21) evaluated at *γ*_th_; that is, *P*_out_ = *P*_*γ*_sel__(*γ*_th_).

Average bit error probability is usually evaluated using the probability of error conditioned over a given SNR in AWGN. This conditional probability of error is defined in terms of standard *Q* function and its average is taken over the PDF of received SNR. Therefore

(23) $$\begin{array}{c} P_{e}=\overset{\infty }{\underset{0}{\int }}P_{e}\left(\frac{\varepsilon }{\gamma_{\text{sel}}}\right)p_{\gamma_{\text{sel}}}\left(\gamma \right)d\gamma , \end{array}$$

where $P_{e}(\varepsilon /\gamma_{\text{sel}})=Q\left(\sqrt{\lambda \gamma_{\text{sel}}}\right)$ and *λ* is a constant and its selection depends on the modulation scheme employed. Referring to the technique in [19], the above equation takes the form

(24) $$\begin{array}{c} P_{e}=\frac{1}{\sqrt{2\pi }}\overset{\infty }{\underset{0}{\int }}P_{\gamma_{\text{sel}}}\left(\frac{t^{2}}{\lambda }\right)e^{-t^{2}/2}dt. \end{array}$$

Solving the above equation using [18, Equation 3.461.2], we obtain

(25) Pe=12Γ(Nj′)×∑J=1M(MJ)J(Pζ)J(1−Pζ)M−J×{1−e−γj′/α∑n=0Nj−11n!(γj′α)n}J−1×{∑k=0∞(−1)kk!(Nj′+k)(2(Nj′+k)−1)!!(λα)(Nj′+k)}.

### 4.2. Best Relay Selection

In order to verify the effectiveness of the proposed multiple relay selection scheme, a similar derivation has been carried out for best relay selection. Based on the same criteria for multiple relay selection, the relay which is able to maximize the SNR of relay-destination link, while satisfying the primary interference threshold, is declared the best relay by the destination. Thus, the optimization problem formulated in (11a) and (11b) can be expressed as

(26) max⁡i′ [γ2i′]s.t. Ii′≤ζ,

where *i*′ denotes the index of the best relay selected for communication.

In order to investigate the system performance for best relay selection, we follow the same assumptions for channel conditions as stated in the above section. Thus, the PDFs and CDFs of *γ*_2*i*_ and *I*_*i*_ will be exponentially distributed as given in (11a) and (11b) for each candidate relay satisfying interference threshold.

Given *K* relays for selection out of *N* potential relays, such that, the interference offered by each *k*th relay is below the interference level *ζ* set by the PU, the conditional PDF of *γ*_sel_, that is, the SNR of the final selected relay, where *γ*_sel_ ∈ *K*, is given according to the proposed relay subset selection scheme as

(27) pγsel(γ ∣ K) =pγ21(γ)Pr⁡[γ21>γ22]⋯Pr[γ21>γ2K]  +pγ22(γ)Pr[γ22>γ21]⋯Pr[γ22>γ2K]  +⋯+pγ2K(γ)Pr[γ2K>γ21]⋯Pr[γ2K>γ2(K−1)].

Assuming the same average values of instantaneous SNRs for all relays to simplify further analysis, (27) can be rewritten as

(28) pγsel(γ ∣ K)=Kpγ2k′(γ)Pr[γ2k′>γ2k]K−1=Kpγ2k′(γ)Pr[γ2k<γ2k′]K−1 k≠k′.

In the above equation, the first part *p*_*γ*_*k*′__(*γ*) is the PDF of SNR of best chosen relay being evaluated at *γ*.

The second part of the equation, that is, Pr[*γ*_*k*_ < *γ*_*k*′_], is the CDF of SNR of *k*th relay being evaluated at *γ*_*k*′_. Since the SNR of each *k*th relay follows the exponential distribution as mentioned above, then the conditional PDF of selected relay using (11a) and (11b) will take the form as given by

(29) $$\begin{array}{c} p_{\gamma_{\text{sel}}}\left(\gamma \;|\;K\right)=\frac{K}{\alpha }e^{-\gamma /\alpha }{\left[1-e^{-\gamma_{2k^{\prime }}/\alpha }\right]}^{K-1}. \end{array}$$

An important consideration is that the PDF obtained in the above equation is conditioned over *K*, that is, the number of relays which satisfies the interference constraints. The value of *K* may take any value from 0 to *N*. If *K* = 0, communication between secondary source-destination pair is not possible. This situation occurs if the relay network experiences a too high interference threshold level set by the PU which is not satisfied by even a single relay, thus making secondary communication impossible. But this is not the case in our scenario as the relay network is assumed to be far away from the PU. Thus, *K* takes the values between 1 and *N*. If *K* = 1, there would be no relay subset selection, and if *K* ≥ 2, the destination picks up the best relay satisfying the proposed criteria. Each member of the potential relay set can satisfy the interference constraint *ζ* with a probability *P*_*ζ*_, where *P*_*ζ*_ = Pr(*I*_*k*_ ≤ *ζ*) dictates the exponential distribution and *P*_*ζ*_ = 1 − *e* ^−*ζ*/*β*^ following the same assumption for interference strengths of relayed links; that is, *β*_1_ = *β*_2_ = ⋯ = *β*_*K*_ = *β*.

Thus, the probability of availability of *K* relays out of *N* relays which satisfy interference threshold follows binomial distribution

(30) $$\begin{array}{c} p_{K}\left(K;N,P_{\zeta }\right)=\left(\begin{array}{c} N\\ K \end{array}\right){\left(P_{\zeta }\right)}^{K}{\left(1-P_{\zeta }\right)}^{N-K}. \end{array}$$

The unconditional PDF of SNR *γ*_sel_ due to the best selected relay can be found by using (11a), (11b), and (30) in (29) as

(31) pγsel(γ;Ik≤ζ)=e−γ/αα×∑K=1N(NK)K(Pζ)K(1−Pζ)N−K×[1−e−(γ2k′/α)]K−1.

The corresponding CDF can be obtained by integrating the above PDF w.r.t. *γ*. The resulting CDF is

(32) Pγsel(γ)=1−e−γ/α×∑K=1N(NK)K(Pζ)K(1−Pζ)N−K×[1−e−γ2k′/α]K−1.

Outage probability can be directly obtained from the CDF of the received SNR given in (32) evaluated at *γ*_th_; that is, *P*_out_ = *P*_*γ*_sel__(*γ*_th_).

Average bit error probability using the same technique as those employed for multiple relay selection and using [18, Equation (3.321.3)] is given by

(33) Pe=12αλ1+αλ/2×[∑K=1N(NK)K(Pζ)K(1−Pζ)N−K           ×[1−e−γ2k′/α]K−1].

In the next section, the results derived for the single best relay selection and multiple relay selection have been investigated for a well-defined range of certain parameters for the primary and secondary networks.

## 5. Simulation Results

This section verifies the effectiveness of the proposed scheme for selecting the subset of relays. For all simulations, source transmit power *P*_*s*_ is set to 10. Zero mean unit variance AWGN is assumed for each link. Furthermore, for the relay subset selection algorithm, *N* and *N*_*j*′_ represent the number of potential relays and selected relays, respectively. Binary phase shift keying (BPSK) with *λ* = 2 is the modulation scheme employed. The interfering channels towards the PU are generated by setting *β* = 0.9*α*.  Table 1 provides the parameter settings for the performance evaluation.

**Figure 3:**
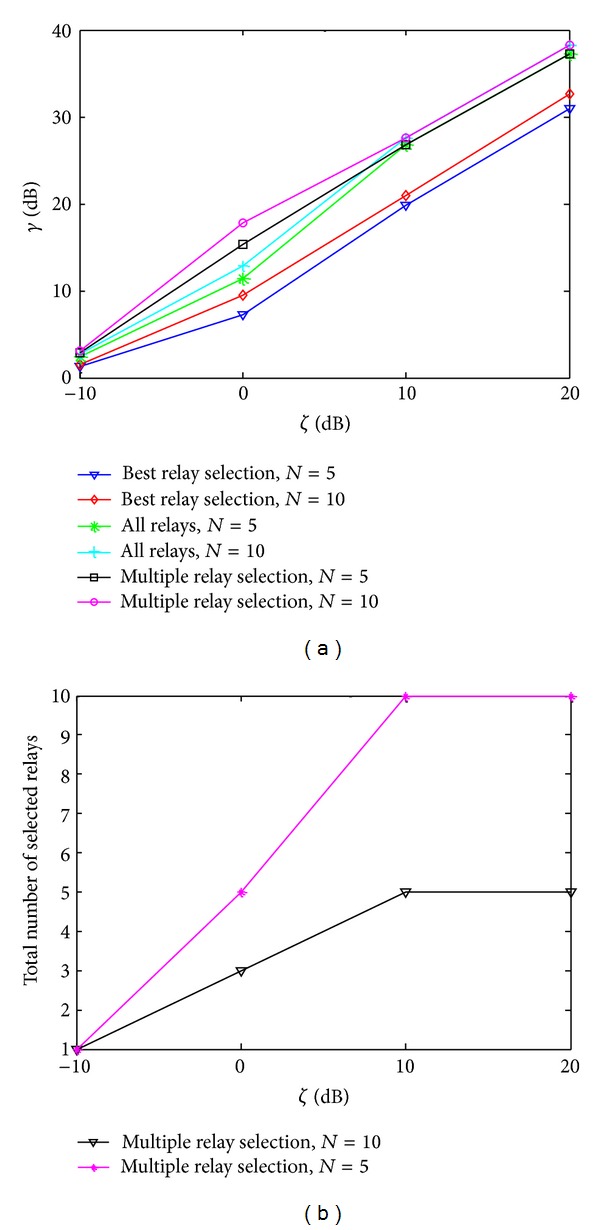
(a) SNR performance versus interference threshold *ζ* and (b) corresponding number of selected relays.

Figure 3 provides the comparison of best relay selection, multiple relay selection, and all relays Participation schemes in terms of SNR achieved at the relay-destination links against different levels of interference threshold *ζ*.  Figure 3(a) shows that the multiple relay selection algorithm outperforms both the best relay selection and all the relay techniques due to freedom of selecting the best subset of relays which can maximize secondary system performance through controlled transmit power allocation to the relay network keeping in view the privilege of PUs. However, in order to allow all relays to participate in transmission, source transmit power needs to be suppressed keeping in view the interference constraint, which in turn produces negative effect on the power received at the relay network, eventually decreasing the SNR received at the destination. Furthermore, a single best relay is also unable to maximize the secondary performance through single best relay. The corresponding total number of selected relays is shown in Figure 3(b). There is a very strong observation that if the interference threshold is made too tight, the multiple relay selection problem reduces to single best relay selection, whereas, on the other hand, relaxing the interference threshold adds more relays to the network, and eventually, maximum cooperative diversity is achieved for *ζ* ≥ 10 dB. Moreover, the greater the number of candidate relays in the potential relay network, the higher the flexibility added to the system to allow more relays to participate in the communication, which are favorable for secondary communication and not harmful for primary communication at the same time, as shown in the case for *ζ* = 0 dB. Thus, the multiple relay selection scheme is the optimal choice for medium levels of interference threshold.

**Figure 4:**
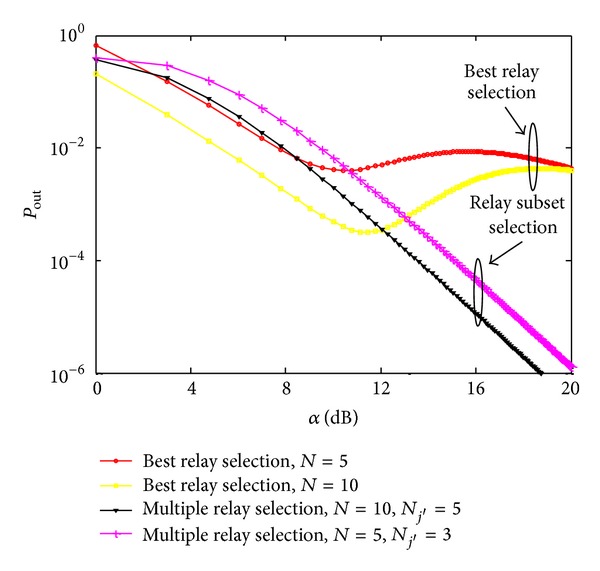
Outage probability with *γ*_th_ = 1 and *ζ* = 1 for different sizes of potential relay network.

**Figure 5:**
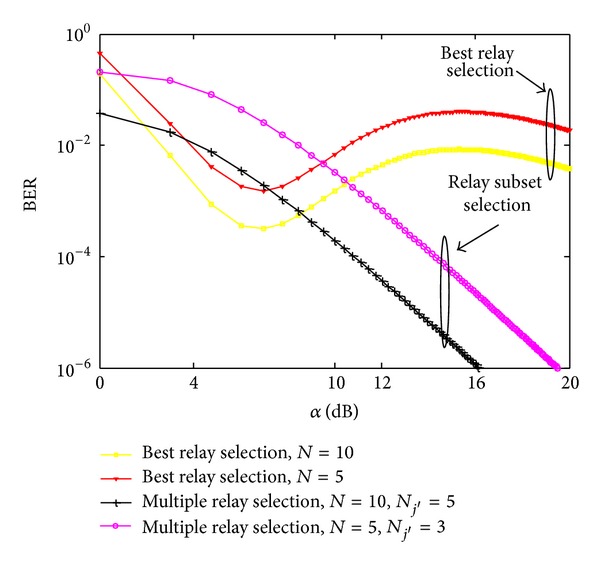
BER with *γ*_th_ = 1 and *ζ* = 1 for different sizes of potential relay network.

In Figures 4 and 5, outage probability and bit error rate of the best and multiple relay selection schemes are investigated, respectively, by varying the average SNR per hop for the different number of potential relays *N*. *ζ* and *γ*_th_ are both set to 1, respectively [20, 21]. As obvious from Figure 4, the outage probability is maximum for the single best relay selection and significantly decreases in the case of proposed relay subset selection due to the fact that spatial diversity enhances system performance by improving SNR received at the destination. An important observation is the improved system performance in the case of proposed multiple relay selection scheme, because in order to design an underlay network with full cooperative diversity, transmit power of the source needs to be suppressed even if the relays just forward the received signal without any further amplification. On the other hand, in multiple relay selection, increasing the number of potential relays generates more subsets which are able to satisfy the interference threshold set by the PU, thus giving more freedom to choose the optimal combination of relays which exhibit good channel conditions towards the destination. Furthermore, relay selection gives priority to those relays that exhibit good channel conditions towards secondary destination and allows them to transmit at high power to improve secondary throughput. Similar trends are observed in Figure 4 due to the same reasons.

## 6. Conclusion

The major contribution of this paper is the derivation of the outage probability and bit error rate for multiple relay selection. For this purpose, a multiple relay selection algorithm is proposed for CRNs operating in an underlay environment near a PU. In this scenario, we select the optimal combination of relays from the potential relay set aiming to maximize the SNR received at the destination, keeping in view the interference threshold of the primary network. The proposed scheme proves the effectiveness of multiple relay selection in energy-constrained CRNs. Finally, the outage probability and average probability of error have been derived in closed forms through the CDF of the received SNR at secondary destination, which has not been done in the literature so far for multiple relay selection. Performance evaluation shows that multiple relay selection outperforms best relay and all relay techniques. Simulation results recommend different operating points for the entire system under different levels of interference threshold and number of potential relays. In future research, this work will be extended to include the line-of sight path between source-destination pair, and also considering the interference from the concurrent primary transmissions.

## Figures and Tables

**Algorithm 1:**
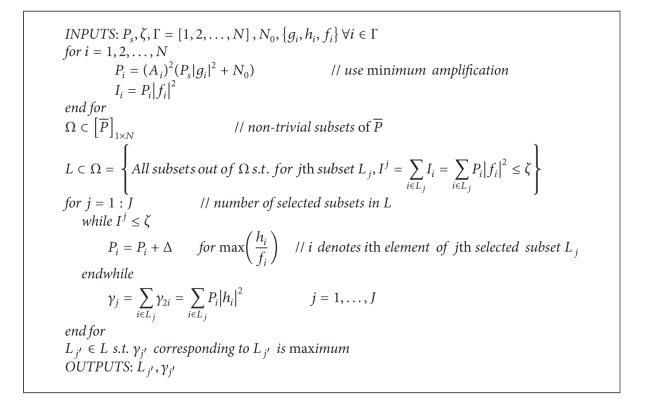
The pseudocode of the proposed algorithm.

**Table 1:** Parameter settings.

Parameters	Values
*g*_*i*_	0.3–0.9
*f*_*i*_	0.1–0.3
*h*_*i*_	0.4–0.9
*N*_0_	1
